# Hyaluronic acid and platelet-rich plasma, a new therapeutic alternative for scleroderma patients: a prospective open-label study

**DOI:** 10.1186/s13075-019-2062-0

**Published:** 2019-12-13

**Authors:** Roberto Pirrello, Barbara Verro, Giulia Grasso, Piero Ruscitti, Adriana Cordova, Roberto Giacomelli, Francesco Ciccia, Giuliana Guggino

**Affiliations:** 1 Dipartimento di Discipline Chirurgiche, Oncologiche e Stomatologiche, Sezione di Chirurgia Plastica e Ricostruttiva, Università di Palermo, Palermo, Italy; 2 Dipartimento di Promozione della Salute, Materno-Infantile, Medicina Interna e Specialistica di Eccellenza “G. D’Alessandro”, Sezione di Reumatologia, University of Palermo, Palermo, Italy; 3 Department of Biotechnological and Applied Clinical Science, Rheumatology Unit, School of Medicine, University of L’Aquila, Palermo, Italy

**Keywords:** Systemic sclerosis, Therapy, Hyaluronic acid, Platelet-rich plasma

## Abstract

**Background:**

Systemic sclerosis is a systemic connective tissue disease characterized by endothelium damage, fibrosis, and subsequent atrophy of the skin. Perioral fibrosis produces a characteristic microstomia together with microcheilia, both of which cause severe difficulties and affects patients’ daily life, such as eating and oral hygiene. Since there are no effective and specific therapies, we have aimed at evaluating the response to filler injections of hyaluronic acid together with platelet-rich plasma.

**Methods:**

Ten female patients aged between 18 and 70 were included in this study. Each patient was treated with three filler injections of hyaluronic acid and platelet-rich plasma at an interval of 15 to 20 days. Follow-up check-ups were recorded 1, 3, and 24 months after the end of the treatment. During the therapy and the subsequent follow-up, we evaluated the mouth’s opening, freedom of movement of the lips, and skin elasticity.

**Results:**

After the treatment, patients had achieved good results already after the first injection and the improvement was maintained in the following months, up to 2 years. In particular, 8 (80%) patients showed a greater mouth’s opening and increased upper lip’s thickness during 1-month follow-up and maintained these results after 2 years (maximum mouth’s opening T0 47.61; T3 49.23; T4 48.60 *p* <  0.0001. Upper lip’s thickness T0 4.20; T3 4.75; T4 4.45 *p* <  0.0001). Moreover, distance between upper and lower incisors (T0 27.05; T3 29.03; T4 28.14 *p* < 0.0001), inter-commissural distance (T0 49.12; T3 51.44; T4 50.31: *p* < 0.0001), and lower lip’s thickness (T0 3.80; T3 4.85, 5.10; T4 4.25; *p* < 0.0001) were increased in all of patients in 1-month follow-up, keeping these benefits after 24 months and having a significant increase of skin elasticity 1 month after the end of therapy.

**Conclusions:**

Our study demonstrates that filler injections of hyaluronic acid and platelet-rich plasma represent an efficient local therapeutic alternative for patients affected by scleroderma. The treatment has significantly improved patients’ quality of living.

## Background

Systemic sclerosis (SSc) is a systemic connective tissue disease characterized by endothelium damage, fibrosis, and subsequent atrophy of the skin, subcutaneous tissue, muscles, and internal organs (i.e., digestive tract, lungs, heart, kidney) [1, 2]. Skin fibrosis is prominent and widespread in diffuse cutaneous SSc (dcSSc), whereas in limited cutaneous SSc (lcSSc) vascular complications rather than fibrosis tend to predominate [1]. Fibrotic skin is characterized by thick dermis and obliteration of hair follicles, sweat glands, and cutaneous blood vessels. Initially, fibrosis is most prominent in the reticular dermis but the subjacent adipose layer will also progressively be affected. These events are responsible for modifying facial features, by showing tight skin with loss of wrinkles and delivering a typical “mask-like” face. Moreover mild to severe restriction of mouth opening is also described [2–4].

Perioral fibrosis produces a characteristic microstomia together with microcheilia, causing severe difficulties in different aspects of daily life, such as eating and oral hygiene. In the end, due to fibrosis, patients lose their facial skin feeling [5].

Skin thickening is an important manifestation of systemic sclerosis affecting patients’ life, with only 2% of patients not reporting this sign.

In consideration of all the above observations, the treatment of skin fibrosis represents one of the major goals for these patients. Unfortunately, skin atrophy therapy remains a main unmet need [3].

Although there are no specific drugs for treatment, many strategies have been tried (pharmacological or non-pharmacological treatment) [3–6] such as UVA phototherapy [7–9], corticosteroids and immunomodulators [10], topical calcitriol [11–13], and etretinate [14–17], but none of these has given a good and long-lasting result.

Recent findings support injections of hyaluronic acid (HA), an anionic, non-sulfated glycosaminoglycan distributed widely throughout connective, epithelial, and neural tissues as a possible treatment of skin fibrosis [18, 19]. Indeed, this could be a valid therapy due to its properties of filling the gaps and softening and moisturizing the skin being able to bind water [20]. Moreover, studies have demonstrated that it induces production of type I collagen in the dermis and this could explain its long-lasting effects [21, 22].

Also, platelet-rich plasma (PRP) [23], a plasma fraction whose concentration is above the peripheral blood level able to release various growth factors [24, 25] that enhance wound-healing, represents a possible effective treatment for the skin ulcers in SSc patients. In particular, these growth factors and their role in cell proliferation, angiogenesis, and inflammation suppression explain PRP use in this new protocol.

In relation to HA and PRP properties, this study shows the results derived by the combined HA and PRP as regenerative treatment for the facial fibrotic skin of SSc patients.

## Material and methods

### Patient data

This prospective, open-label, monocentric single-arm study was conducted in patients with systemic sclerosis recruited from the Rheumatology Section of Policlinico “Paolo Giaccone” of Palermo.

In this study, we enrolled 10 patients with SSc diagnosed according to the 2013 classification criteria for systemic sclerosis [26–30]. Patient characteristics are described in Table 1. Modified Rodnan skin score was calculated to assess the extension of skin involvement [31].

**Table 1:** Demographic and clinical characteristics of patients

	Gender	Age	SSc type	Disease duration	Autoantibodies	DMARDs
Patient 1	F	38	Limited	7	ANA, anti-CENP-B	MMF, HCQ
Patient 2	F	62	Limited	7	ANA	RTX
Patient 3	F	53	Diffuse	4	ANA, anti-Scl70	AZA, HCQ
Patient 4	F	47	Diffuse	8	ANA, anti-Scl70	RTX
Patient 5	F	38	Diffuse	2	ANA, anti-Scl70	AZA
Patient 6	F	45	Diffuse	9	ANA, anti-CENP-B	HCQ
Patient 7	F	33	Diffuse	9	ANA	MMF
Patient 8	F	51	Diffuse	5	ANA, anti-Scl70	MMF
Patient 9	F	41	Diffuse	2	ANA	HCQ
Patient 10	F	40	Diffuse	7	ANA, anti-Scl70	MMF, HCQ

*MMF* mycophenolate mofetil, *HCQ* hydroxychloroquine, *RTX* rituximab, *AZA* azathioprine

This study was approved by the Ethical Committee of the University Hospital in Palermo, and informed consent was obtained from each patient in accordance with the Helsinki Declaration.

The study exclusion criteria were as follows: the presence of infectious disease (i.e., HIV, HBV, HCV), pregnancy, lactation, and BMI less than 17 kg/m^2^.

### A-CP HA KIT medical device

A-CP HA Kit is a medical device used for the extemporaneous production of a mixture of platelet-rich plasma and hyaluronic acid with a closed-circuit procedure to ensure sterility. In this kit, there are three tubes A-CP HA sterile, apyrogenic, and disposable, individually blistered, to be used in three different sessions.

Each A-CP HA tube is vacuum and contains:

- Two milliliters of HA gel in phosphate buffer, obtained by fermentation, that it is linear, non-cross-linked, medium-high molecular weight.
- Two milliliters of inert polyester separator gel. It is a latest-generation thixotropic gel, covered by an international license, biocompatible, for clinical use, which ensures an optimal and standardized cellular recovery in the PRP and separates these cells from the waste component, reducing contamination risk and making the technique non-operator sensitive.
- 0.6 ml of anticoagulant (sodium citrate 4%).

**Fig. 1:**
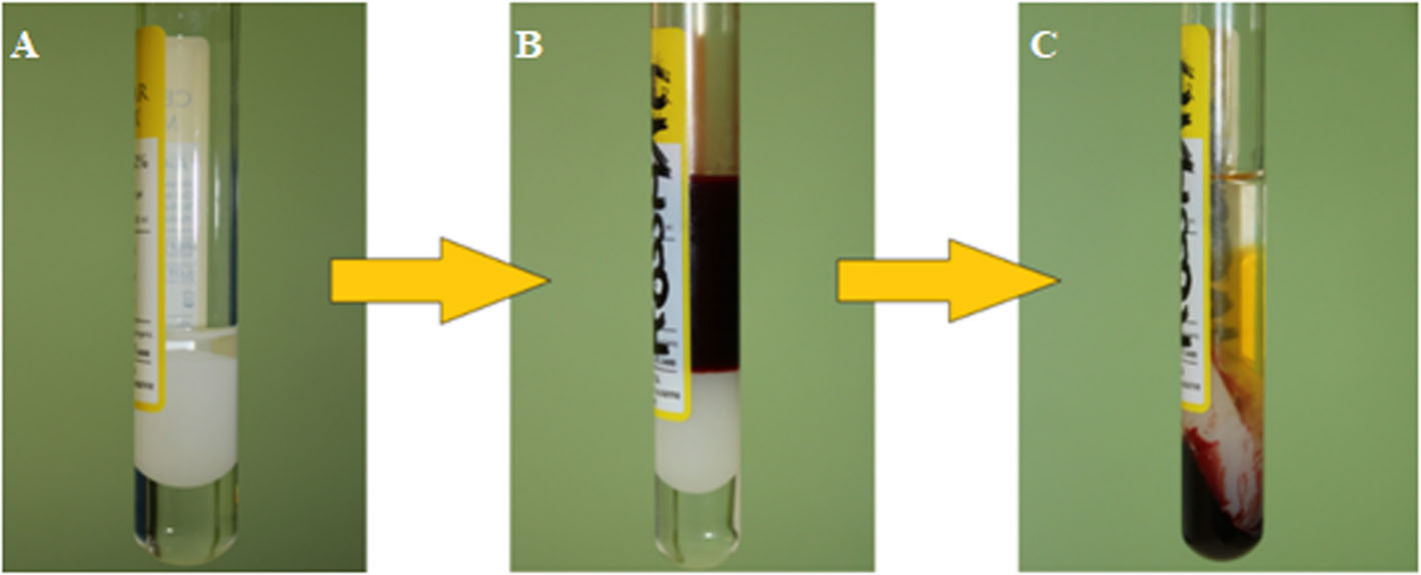
A-CP HA medical device: tube containing 2 ml of HA gel in phosphate buffer, 2 ml of inert polyester separator gel, and 0.6 ml of anticoagulant (**a**), tube with 7 ml of blood (**b**), and tube after centrifugation at 1500 g for 5 min (**c**)

Briefly, the PRP and HA were obtained by mixing 7 ml of blood taken from a peripheral vein and automatically collected in the tubes. Then, the tube was centrifuged at 1500*g* for 5 min with one-step technique. After the centrifugation, we obtained about 3 ml of PRP—the concentration may be different from patient to patient in accordance with blood platelet concentration—with platelet recovery over 80% and white and red cells depletion over 90% and 99.5% respectively. Later, we rolled the tube horizontally in order to obtain a homogeneous mixture of PRP and HA without damaging cells. The final result of this process was a mixture of about 4 ml of PRP and HA in the same proportion (2 ml of PRP and 2 ml of HA) that was distributed on 1-ml Luer-Lock syringes with 27-G needle (Fig. 1).

### Videodermatoscopy

Before the treatment, skin patients were examined by videodermatoscopy [32] at the Dermatology Section of Policlinico “Paolo Giaccone” of Palermo. In particular, capillaries in the living skin above the left upper lip were examined, and a surface of 1 cm^2^ was taken in each image, applying × 30 and × 150 magnifications, and using white light and fluorescent light.

Another videodermatoscopy was performed about 30 days after the third injection to seek if any changes had occurred during the treatment.

### Skin Elastometer device

During the first medical examination, skin elasticity of patients’ face was measured with *Skin Elastometer* device. The probe was placed on their left cheek and above the left upper lip. After placing the probe, the skin was aspirated for 3 s with a negative pressure of 400 mbar, and at the end of this time, other 3 s was needed for the skin to return to its initial position. The value, shown as a percentage, has to be related to age patient as the elasticity decreases with increasing age. This examination was also done, in the same way, about 30 days after the last injection to seek whether any changes occurred during the treatment.

### Study protocol

**Fig. 2:**
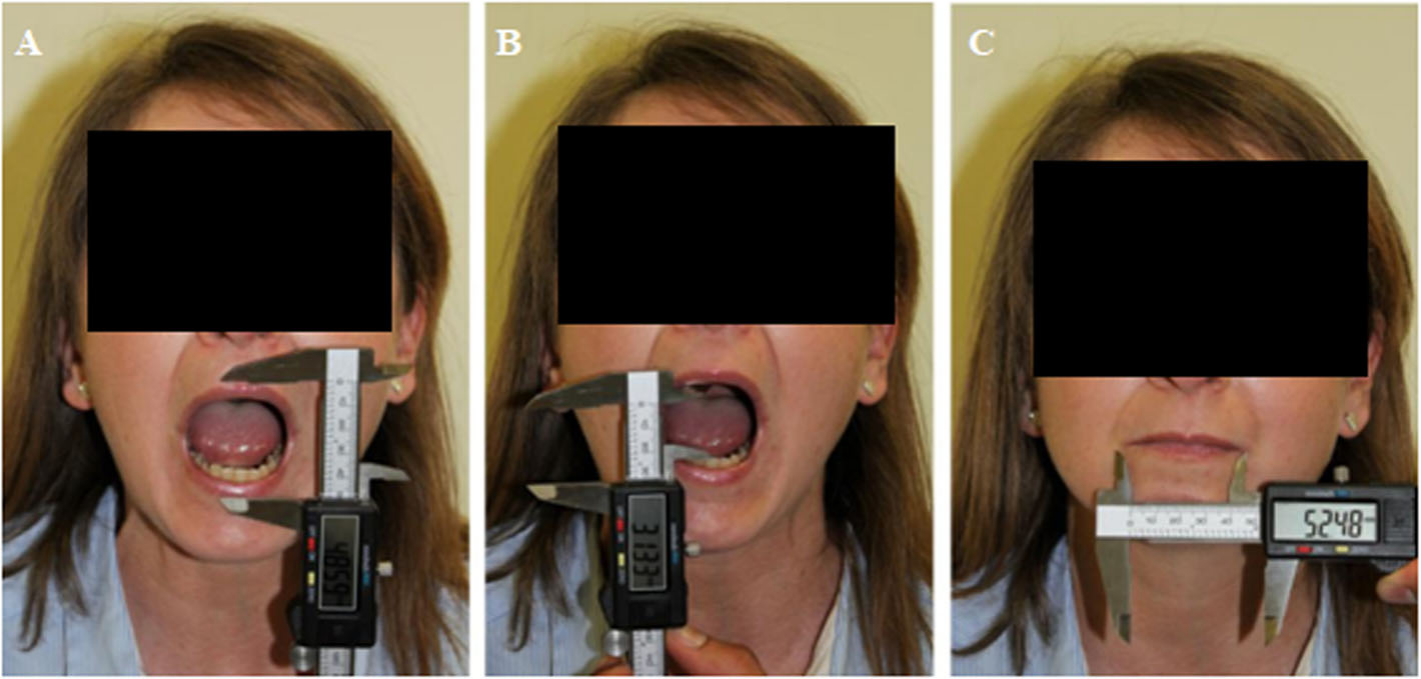
Parameters examined by digital electronic caliper: maximum mouth’s opening (**a**), distance between upper and lower incisors (**b**), and inter-commissural distance (**c**)

Study protocol consists in three injections: each one was performed 15–20 days after the other. The same day of the injection, some parameters were examined using digital electronic caliper (Fig. 2): the maximum mouth’s opening (measured from the vermillion edge of the upper lip to the one of the lower lip), the distance between upper and lower incisors, the inter-commissural distance (measured from a labial commissure to the other, along the vermillion edge of the lower lip with occluded teeth), and the upper and lower lip’s thickness.

Moreover, photos of patients were taken in three different projections—frontal, ¾ right and ¾ left—before and after each injection.

About 15 min before the injection, anesthetic cream was used on the patient’s face to reduce pain. When the mixture of PRP and HA was ready, we gave the injections in temporal and zygomatic regions, nose-lip junction, perioral area, lips, and chin. At the end of each treatment, an antibacterial cream based on gentamicin was used to prevent infection and an appropriate cream was used for bruises, when necessary.

Injections were given in the clinic, and they did not cause side effects except some bruises. At the end of each treatment, patients came back home with no pain or discomfort. Thus, there was no need to admit or to observe patients.

When the whole treatment ended, patients were visited again 1, 3, and 24 months after the last injections in order to examine the same parameters: maximum mouth’s opening, distance between upper and lower incisors, inter-commissural distance, and upper and lower lip’s thickness. Moreover, photos of patients were taken in the same three projections: frontal, ¾ right, and ¾ left. On the same day, skin elasticity and capillaries in the living skin were examined as for the first time.

Follow-up ended with an interview with the patients, to know esthetic and functional benefits gained after the treatment, as well as any critical issues and their overall satisfaction about results achieved. Questions about skin hydration degree and need to apply moisturizers, skin elasticity and pain with mouth movements were asked.

### Statistical analyses

Measurements are expressed as mean ± standard deviation (DS). To analyze differences between outcomes measured at different time period, we used both parametric and non-parametric analysis.

Parametric analysis has been conducted using standard one-way repeated measures ANOVA with the Greenhouse-Geisser correction factor to correct for violation of the sphericity assumption. The latter is tested by using the Mauchly test.

Non-parametric analysis has been implemented using the Friedman test. The null hypothesis for the Friedman test is that there are no differences between the outcomes measured at different time period. Rejection of the null-hypothesis indicates that that two or more outcome’s time-specific measures are significantly different from each other.

Finally, to investigate whether treatment is persistent, we also rely on the non-parametric Wilcoxon signed-rank test which compare mean ranks of each period with the baseline measured at time 0.

*p* < 0.05 was considered significant.

## Results

### Clinical evaluation

**Fig. 3:**
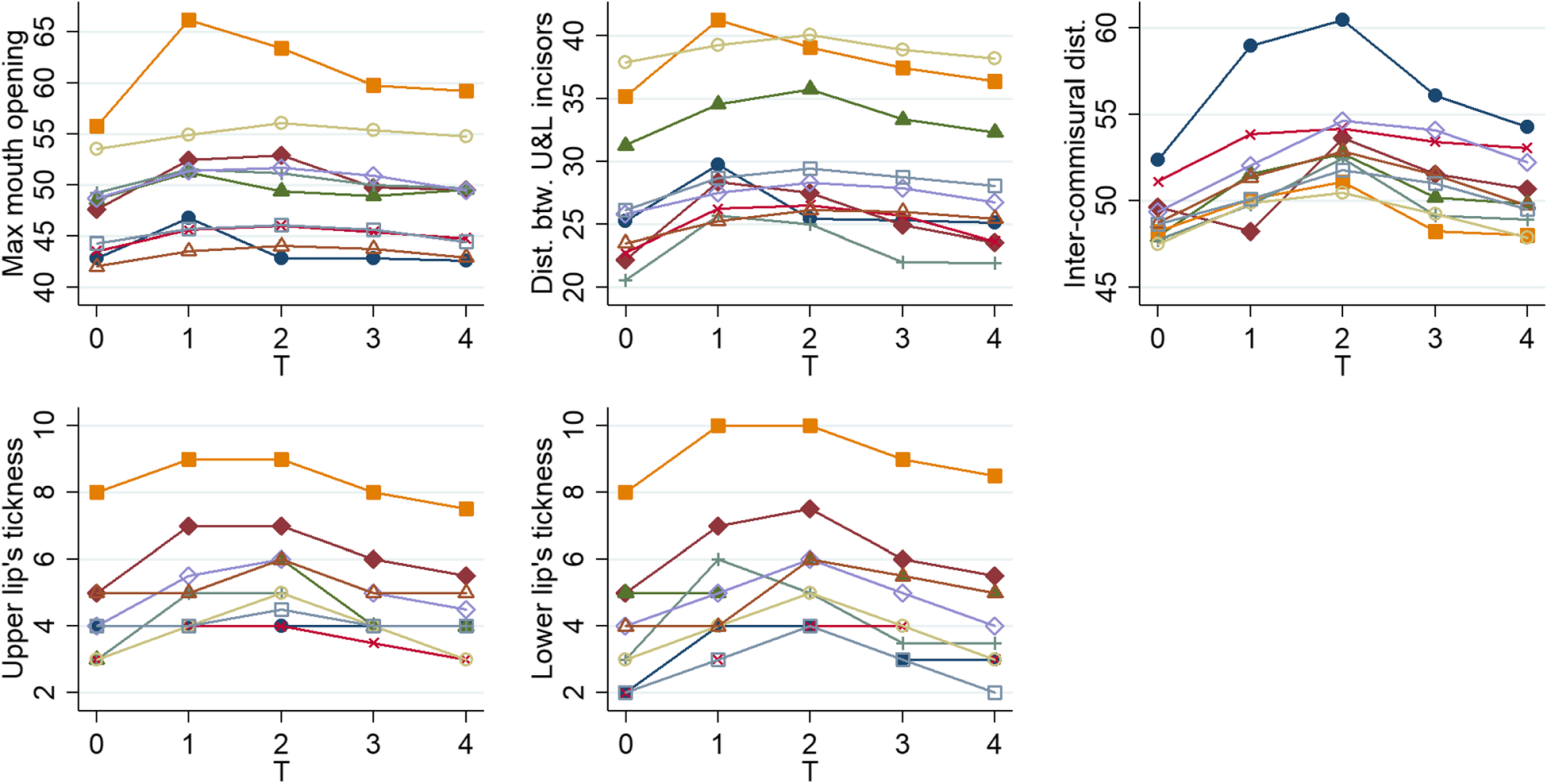
Graphs of clinical parameters (maximum mouth’s opening, distance between upper and lower incisors, inter-commissural distance, upper and lower lip’s thickness) in all patients (each line represents a different patients) for each time point (baseline (T0), during the third injection (T1), the 1-month follow-up (T2), the 3-month follow-up (T3), and the 24-month follow-up (T4))

Clinical parameters were evaluated at each time point, and results are summarized in Table 2 and Fig. 3. The parameters measured during the third injection and during follow-up (1 month, 3 months, and 2 years after the third injection) were compared with the baseline data (before treatment) and all of them showed a statistically significant improvement, although it seems to decline over time. Anybody was lost to follow-up.

**Table 2:** Clinical parameters (maximum mouth’s opening, distance between upper and lower incisors, inter-commissural distance, upper and lower lip’s thickness), expressed as mean ± SD at baseline (T0), during the third injection (T1), the 1-month follow-up (T2), the 3-month follow-up (T3), and the 24-month follow-up (T4)

	Baseline (T0)	1 month (T1)	2 months (T2)	3 months (T3)	24 months (T4)	Comparison to the baseline		ANOVA		Friedman test	
	Mean (SD)	Mean (SD)	Mean (SD)	Mean (SD)	Mean (SD)	*p* value		*F*	*p* value	Test Stat. Appr.	*p* value
						T0/T3	T0/T4				
Maximum mouth’s opening (mm)	47.61 (4.56)	50.91 (6.47)	50.35 (6.19)	49.23 (5.27)	48.60 (5.30)	0.0059	0.0093	11.48	0.0015	30.45	< 0.0001
Distance between upper and lower incisors (mm)	27.05 (5.80)	30.65 (5.71)	30.41 (5.90)	29.03 (5.65)	28.14 (5.61)	0.0051	0.0069	22.36	< 0.0001	33.04	< 0.0001
Inter-commissural distance (mm)	49.12 (1.55)	51.54 (3.01)	53.43 (2.79)	51.44 (2.47)	50.31 (2.02)	0.0058	0.0065	23.68	< 0.0001	32.50	< 0.0001
Upper lip’s thickness (mm)	4.20 (1.54)	5.25 (1.62)	5.65 (1.52)	4.75 (1.35)	4.45 (1.32)	0.0154	0.15	19.64	< 0.0001	29.52	< 0.0001
Lower lip’s thickness (mm)	3.80 (1.87)	5.10 (2.13)	5.75 (1.87)	4.85 (1.81)	4.25 (1.85)	0.0041	0.0163	22.98	< 0.0001	31.15	< 0.0001

### Dermoscopic evaluation

Comparing images obtained at baseline and 30 days after the third injection, we observe that capillary density remained stable in 100% of patients. Vascular ectasia is slightly increased in 4 (40%) out of 10 patients, it has remained stable in 3 (30%) out of 10 patients, and it is undetectable in 3 (30%) out of 10 patients.

### Elastometer evaluation

Comparing measurements obtained before the treatment and 30 days after its end, we can observe that 100% of the patients had a significant increase of skin elasticity (Table 3).

**Table 3:** Skin elasticity on the left cheek and above the left upper lip measured with Skin Elastometer device before the beginning (T0) and 1 month after the end of treatment (T2) (expressed as mean ± SD)

	Baseline (T0)	2 months (T2)	T_2_ − T_0_ (CI 95%)	*p* value
	Mean (± SD)	Mean (± SD)		
Left cheek (%)	58 (± 2.12)	61 (± 2.00)	3 (2.12; 3.88)	0.0007
Left upper lip (%)	60 (± 2.12)	64 (± 2.00)	4 (2.48; 5.52)	0.0019

### Patient’s evaluation

During the therapy and the subsequent follow-up, we interviewed patients we wanted to know about the esthetic and functional benefits derived after the treatment, as well as any critical issues and their satisfaction about results achieved. In particular, all of the patients were satisfied of the improvements. Indeed, in four (40%) cases, the skin became more hydrated and softer with less need to apply moisturizers. Lips had the same outcome. Three (30%) patients have reported increased skin elasticity. Seven (70%) patients said to have regained the feeling of their own skin and skin sensitivity which they had lost years before. Moreover, four (40%) patients could open their mouth and suffered less when doing this movement. Three (30%) patients said that flushing and hematoma (potential side effects of injections) decreased gradually during the treatment.

## Discussion

Systemic sclerosis is a chronic connective tissue inflammatory disease responsible for severe facial skin alterations that cause limitations and difficulties in different areas of daily life, as well as disfiguring malformations and changes in appearance [5]. Additionally, to date, there are no effective local therapies that can guarantee a satisfactory result neither from an esthetic nor from a functional point of view [6–17]. To date, the only therapeutic strategies that achieved results consisted in autologous fat grafting [33, 34]. In particular, some studies are concerned with only the treatment of microstomia using autologous fat grafting with [35] or without PRP [36]. Moreover, Virzì et al. treated sclerodermic facial skin lesions by autologous fat grafting and PRP [37] and Scuderi et al. by a combination of adipose-derived stromal cells (ASCs) in HA solution [38]. Although all of these therapeutic approaches achieved goods results, they show some limitations. In fact, this invasive procedure requires general anesthesia, the use of the operating room, and observation of the patient for many hours. Moreover, only a rate of grafted fat is able to survive depending on several factors that cannot be quantified. By contrast, HA and PRP are produced with a closed-circuit procedure to ensure sterility, injections are given in the clinic, and there is no need to admit or to observe patients after the treatment. Therefore, as far as we know, this study attempted to demonstrate, for the first time, the effectiveness of local intradermal infiltrations of HA and PRP together in the treatment of facial skin fibrosis of SSc patients. Indeed, in other studies, hyaluronic acid has been used as a dermal filler in the treatment of a particular clinical variant of localized scleroderma, the so-called *en coupe de saber* [20, 21, 38, 39], but there are no evidences on the use of platelet-rich plasma when treating this disease.

Therefore, considering HA and PRP properties and their wide and valid use as filler in the treatment of skin lesions of several origin [22, 39–42], in this study, we used HA and PRP infiltrations to treat local facial scleroderma. Indeed, studies have demonstrated that hyaluronic acid fills the gaps and softens and moisturizes the skin for its capacity to bind water [20]. Moreover, it induces production of type I collagen in the dermis, and this could explain its long-lasting effects [21, 22]. Instead, platelet-rich plasma is rich in growth factors that are involved in cell proliferation, angiogenesis, and inflammation suppression that explain PRP use in this study [23–25]. On these bases, our study showed the results derived by their combining as regenerative treatment for the facial fibrotic skin of SSc patients. In fact, the infiltration had allowed to improve not only the esthetic appearance, but also the functional state of the face of the patients, who were able to enjoy re-elastic skin, increased the mouth opening and no limit lip movement, as well as recovering the perception of having the skin on the face. All of this has significantly improved their life quality as they resumed naturally gestures and activities commonly considered automatic and gained more confidence. Our results testify that this treatment gave clinical and functional benefits and this outcome has remained stable over time: improvements achieved were maintained after the end of the treatment although decreased gradually during the following 24 months.

In fact, results have also demonstrated a long retention rate since 60% of patients showed a greater mouth’s opening and increased upper lip thickness and 80% of them showed increased distance between upper and lower incisors, inter-commissural distance, and lower lip thickness up to 2 years later (nobody was lost to follow-up). However, this therapy has limitation as its effect is not permanent [43], but it inevitably requires further infiltrations over time. In fact, although the persistence of the benefit effects lasted after 2 years, results steadily decreased, thus requiring ongoing injections to maintain the outcome, in accordance with other studies [20, 37]. Moreover, our study’s sample consisted of only 10 patients, and consequently, it would be necessary to increase the sample in order to confirm our results and to introduce this protocol as a new therapy.

## Conclusions

Finally, this study has shown the efficacy of hyaluronic acid and platelet-rich plasma infiltrations in the treatment of facial skin lesions in SSc patients. In fact, it has guaranteed a satisfactory result both from an esthetic and a functional point of view, not only visually but also instrumentally observed.

These treatments could be considered as a starting point of a regenerative therapy in SSc patients even if the small sample size will require to confirm our results in a largest cohort of patients.

## Data Availability

All the data are maintained in the Dipartimento di Discipline Chirurgiche, Oncologiche e Stomatologiche, Sezione di Chirurgia Plastica e Ricostruttiva, Università di Palermo.

## Abbreviations

SSc: Systemic sclerosis
HA: Hyaluronic acid
PRP: Platelet-rich plasma
dsSSc: Diffuse cutaneous SSc
lcSSc: Limited cutaneous SSc
ASCS: Adipose-derived stromal cells
MMF: Mycophenolate mofetil
AZA: Azathioprine
HCQ: Hydroxychloroquine
RTX: Rituximab
